# Toward the personalized and integrative management of voriconazole dosing during COVID-19-associated pulmonary aspergillosis

**DOI:** 10.1186/s13054-021-03568-8

**Published:** 2021-04-20

**Authors:** Brendan Le Daré, Christelle Boglione-Kerrien, Florian Reizine, Jean-Pierre Gangneux, Astrid Bacle

**Affiliations:** 1 Centre Hospitalier Universitaire de Rennes, Service Pharmacie, 35000 Rennes, France; 2 INSERM, INRAE, CHU Rennes, Institut NuMeCan (Nutrition, Metabolisms and Cancer), Réseau PREVITOX, Univ Rennes, Rennes, France; 3 Department of Clinical and Biological Pharmacology and Pharmacovigilance, Pharmacoepidemiology and Drug Information Centre, Rennes University Hospital, 35033 Rennes, France; 4 Service des Maladies Infectieuses et Réanimation Médicale, CHU Rennes, 35033 Rennes, France; 5 Faculté de Médecine, Biosit, Université Rennes 1, 35043 Rennes, France; 6 Inserm, EHESP, Irset (Institut de Recherche en Santé, environnement et travail) - UMR_S 1085, CHU Rennes, Univ Rennes, 35000 Rennes, France

COVID-19-associated pulmonary aspergillosis (CAPA) has raised concerns about increased mortality, and a consensus has been recently prepared to define and manage this pathology. Voriconazole is currently recommended as first-line therapy for CAPA [1]. Voriconazole is a second-generation triazole antifungal agent extensively metabolized via cytochrome P450 (CYP450) isoenzyme CYP2C19 and, to a lesser extent, CYP2C9 and CYP3A4. Voriconazole exhibits highly variable nonlinear pharmacokinetics and a narrow therapeutic range. In addition, several conditions of critical-care unit patients, including impaired renal or hepatic function, continuous renal replacement therapy, or extracorporeal membrane oxygenation and altered volume of distribution, render plasma drug concentrations difficult to predict, making therapeutic drug monitoring (TDM) a potential key component in the treatment of CAPA. Furthermore, severe COVID-19 patients often receive multiple drugs, increasing the risk of drug–drug interactions [1]. There is growing evidence that the inflammatory state can also alter the metabolism and thus the pharmacokinetics of many drugs [2]. Given the seriousness of the inflammatory states that occur in COVID-19, particularly during “cytokine storms” and the introduction of corticosteroids as an early and efficient strategy, it is essential to study their clinical and biological impact. We observed unexpected variations of voriconazole plasma concentrations in COVID-19 patients hospitalized in an intensive care unit (ICU). In the case reported in Fig. 1, after introduction of voriconazole for ARDS-associated CAPA, inflammation markedly influenced the voriconazole trough concentration (VTC). Metabolite ratios (voriconazole N-oxide /voriconazole) were < 0.3 during the inflammatory period and > 1 outside the inflammatory period. In addition, the time during which C-reactive protein (CRP) decreased sharply (from 200.9 to 46.8 mg/L over four days) corresponds to the period of a sharp decrease in the VTC, suggesting a recovery of metabolic function.

**Fig. 1:**
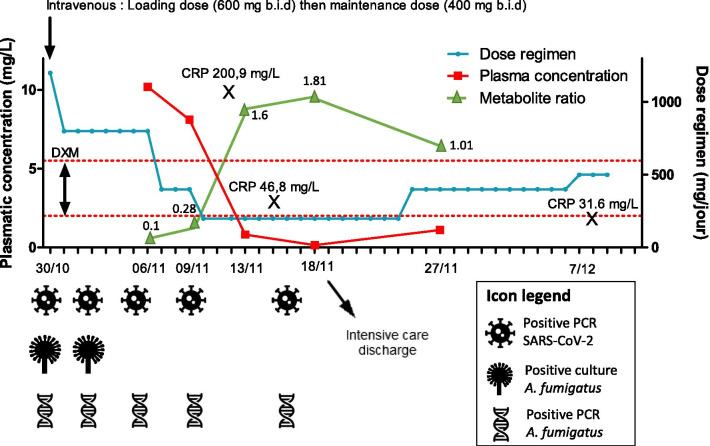
Biological findings for a COVID-19 patient treated with voriconazole in the context of pulmonary aspergillosis. *MR* metabolite ratios (voriconazole N-oxide /voriconazole), *DXM* dexamethasone. The dose regimen of voriconazole is depicted in blue, the voriconazole trough plasma concentration in red, and MR in green. Voriconazole was not administered on the evening of November 10, 2020, nor the morning or evening of November 11, 2020

We propose an empirical algorithm to optimize the management of measuring VTC of CAPA patients to avoid misdosing (Fig. 2). First, monitoring of inflammation, at least by measuring CRP, is essential before initiation of voriconazole and then regularly to follow its evolution. As CRP levels above 96 mg/L have been identified as an independent risk factor for voriconazole overdose among hematological patients (Odds Ratio 27; IC 95% [6–106]), we propose to consider reducing maintenance doses of voriconazole if the CRP level exceeds this threshold [3]. Second, co-administration of other drugs must be carefully followed, first because of the impact of anti-inflammatory drugs, such as corticosteroids, on the inflammatory process, as well as because of potential drug-drug interactions. Because voriconazole is metabolized via CYP2C19, CYP2C9, and CYP3A4, it is among the drugs that are the most frequently associated with major drug–drug interactions in the ICU setting [1]. Dexamethasone is now widely used in COVID patients and induces CYP2C9, which could decrease the VTC [4]. In addition, proton-pump inhibitors widely used in the ICU are known to inhibit CYP2C9, CYP2C19, and CYP3A4, and can thus increase the VTC, except for pantoprazole [5]. Lastly, voriconazole TDM is essential for ICU patients, particularly COVID-19 patients, with the aim to maximize efficacy and minimize toxicity. For these reasons, VTC should be performed more frequently while the CRP remains above 96 mg/L, but also when inflammation decreases. Further studies would be needed to assess whether changes in voriconazole protein binding occur during the inflammatory period, as shown for lopinavir [6].

**Fig. 2:**
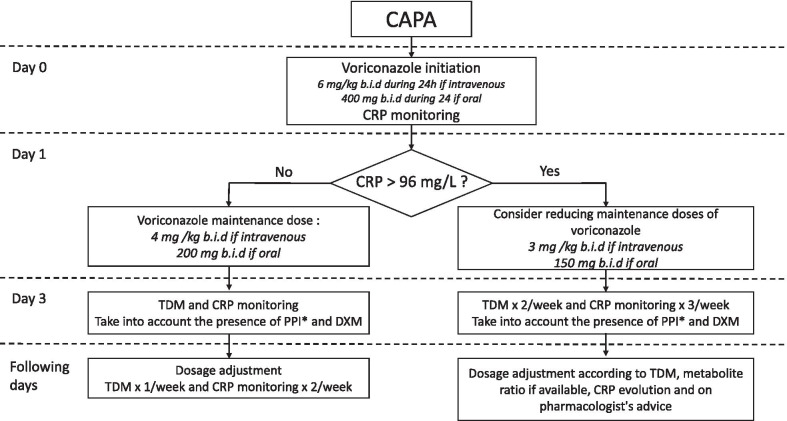
Empirical algorithm for voriconazole dose adjustment in CAPA patients. *CAPA* COVID-19-associated pulmonary aspergillosis; *PPI* proton pump inhibitor; *DXM* dexamethasone; *TDM* therapeutic drug monitoring; *CRP* C-reactive protein; *b.i.d* twice a day; *: except for pantoprazole

Overall, these findings suggest that therapeutic management must take into account the high level of inflammation of CAPA patients. As it is based on parameters that are readily available in comparison with VTC, the empirical algorithm presented here, that must consider clinical and biological parameters of each patient, could be considered with the goal of improving patient management.

## Data Availability

The datasets used and/or analyzed during the current study are available from the corresponding author on reasonable request.

## Abbreviations

CAPA: COVID-19-associated pulmonary aspergillosis
CYP450: Cytochrome P450
DXM: Dexamethasone
PPI: Proton pump inhibitor
TDM: Therapeutic drug monitoring
VTC: Voriconazole trough concentration
